# Acute Bilateral Traumatic Achilles Tendon Rupture – A Rare Presentation

**DOI:** 10.7759/cureus.706

**Published:** 2016-07-20

**Authors:** Chirag Kapoor, Maulik Jhaveri, Paresh Golwala, Aditya Merh, Amit Patel

**Affiliations:** 1 Orthopaedics, Sumandeep Vidyapeeth, Vadodara, Gujarat

**Keywords:** tendoachilles, rupture, bilateral, ultrasonography

## Abstract

The Achilles tendon is the strongest tendon in the body, which is commonly ruptured in male athletes. Bilateral rupture of the Achilles tendon is a rare condition with very few reported cases in the literature. It poses a challenge in management, and hence, we report a case with traumatic bilateral Achilles tendon rupture in a young male patient and its management. One side was treated conservatively as the rupture was partial and the other side, which had a complete tear, was operated. At nine months follow-up, the patient has had a satisfactory result and is now bearing full weight without any problems. We suggest this method of treatment to be worthwhile for this unusual entity.

## Introduction

The Achilles tendon is the strongest tendon in the body [1]. It can bear up to 12 times of the body weight and accounts for 20% of all large tendon ruptures [2]. It is the most commonly ruptured tendon of the lower limb and primarily affects men of the age group 30 - 50 years [3].

Normally, Achilles tendon consists of mainly Type-I collagen. Sometimes Type-III collagen is seen which is less resistant to tensile forces and predisposes the tendon to rupture [4].

The tendon is usually torn either as a result of a large force or as a result of physiological force if it is weakened. Most of the tears occur in the watershed area, which is an area of structural weakness approximately 6 cm proximal to the tendon insertion on the calcaneus. The most common cause of Achilles tendon rupture is trauma during sports [5].

Bilateral Achilles tendon ruptures are rare with an incidence of about 1% [6].

These injuries are difficult to deal with and because the reported incidence is less in literature, we present a rare case of acute bilateral traumatic Achilles tendon rupture in a young male patient and its management.

## Case presentation

A 37-year-old male suffered an injury while playing badminton. The patient heard a snap in both the ankles after which he could not get up and was unable to walk.

The patient came to us 10 days after the injury with pain over the retrocalcaneal region in both ankles. On clinical examination, there was swelling, tenderness, and a palpable gap over the retrocalcaneal region. The calf squeeze test (Thompson test) was positive; i.e. no ankle plantar flexion was present on squeezing the calf and the heel raise test was negative.

The patient had no history of any medical illness. His routine blood investigations were normal. Informed patient consent was obtained for treatment.

**Figure 1:**
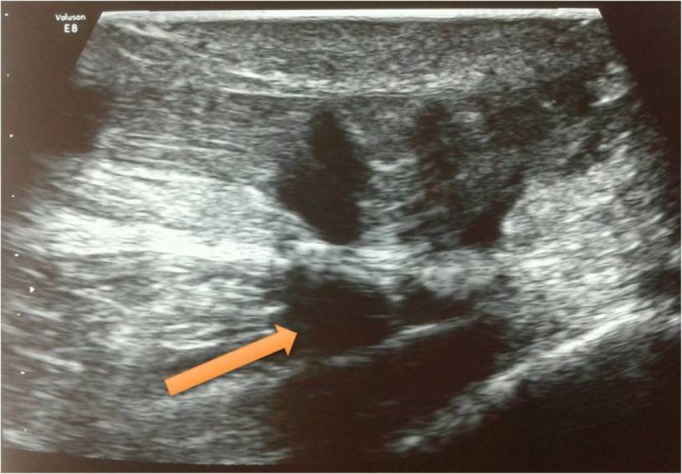
Ultrasonography of right Achilles tendon showing complete rupture

**Figure 2:**
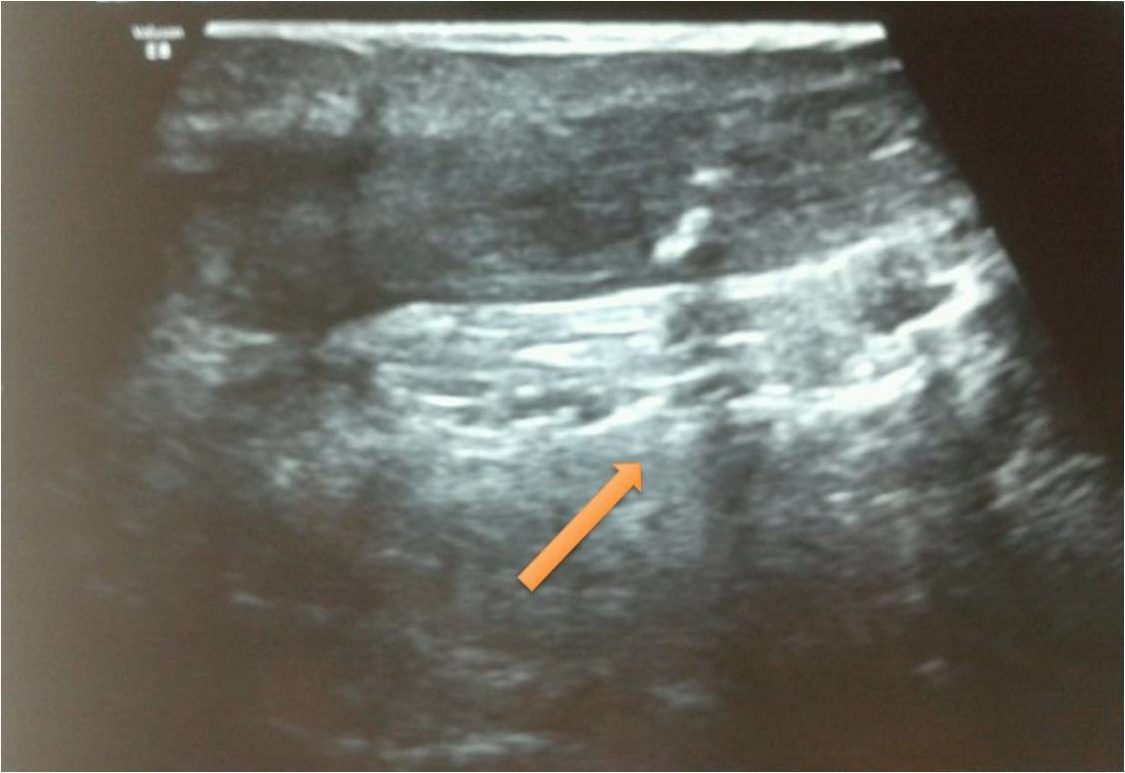
Ultrasonography of left Achilles tendon showing partial rupture

Real-time ultrasonography of both the ankles was done which showed a complete tear of the Achilles tendon on the right side (Figure 1) and a partial tear on the left side (Figure 2).

The tear on the left side was treated conservatively by an above-knee plaster cast with the ankle in plantar flexion for one month. On the right side, an end-to-end repair was done with Ethibond sutures with anchorage in calcaneum and an above-knee plaster cast was applied in plantar flexion for one month.

After one month, a below-knee plaster cast was applied on both limbs. At eight weeks, the cast was removed, ankle movement was started, and the patient was allowed to begin walking full weight-bearing.

**Figure 3:**
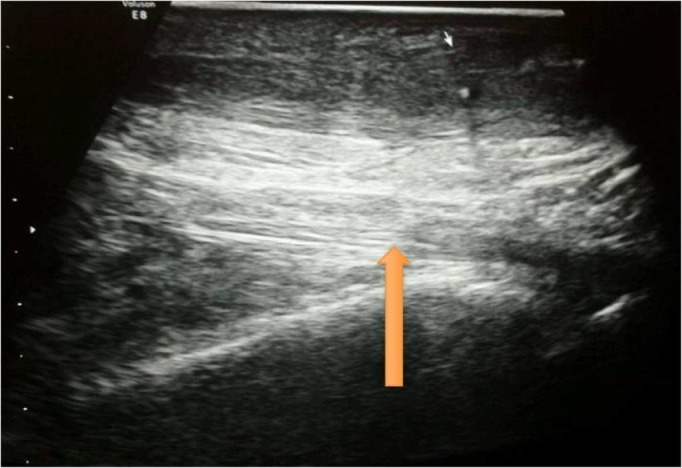
Ultrasonography of right Achilles tendon at nine months follow-up showing complete healing of the tendon.

**Figure 4:**
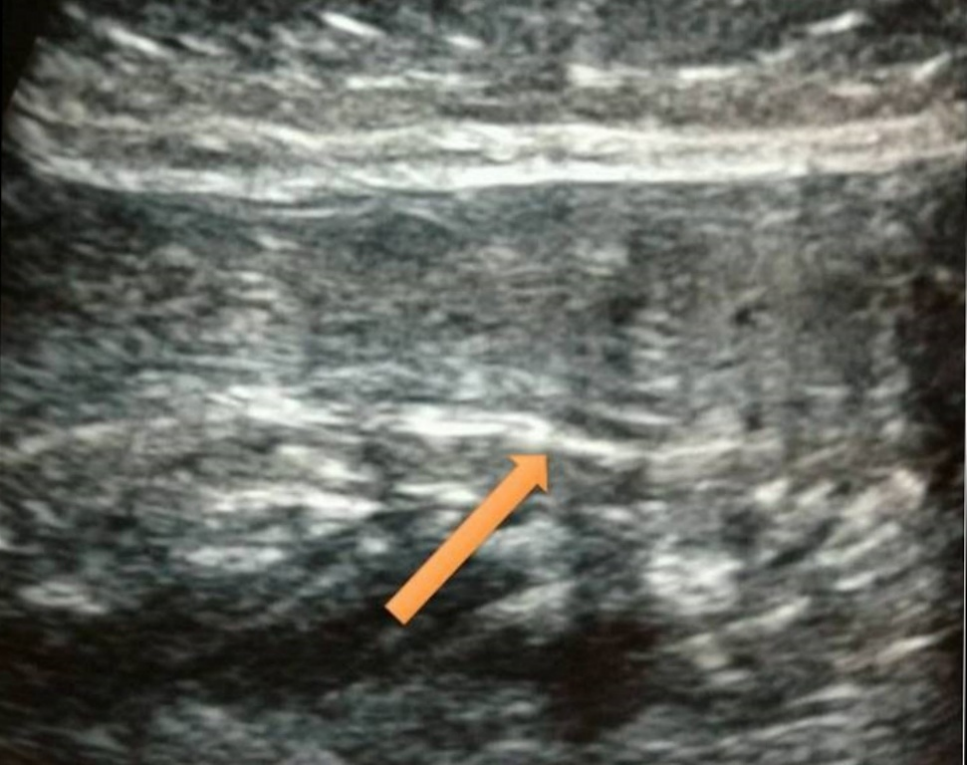
Ultrasonography of left Achilles tendon at nine months follow-up showing complete healing of the tendon.

At his nine-month follow-up, the tendon continuity was intact both clinically and radiologically, and the patient has been able to ambulate full weight-bearing (Figures 3-4).

## Discussion

Hippocrates was the first to describe injury of the Achilles tendon. Bilateral Achilles tendon rupture is rare and few cases have been documented in the literature. Factors that predispose individuals to these types of injuries include long-term corticosteroid therapy, diabetes, xanthomatosis, chronic renal failure, and use of ﬂuroquinolones [7].

In 2004, Hayes, et al. reviewed all published cases of bilateral Achilles tendon rupture and found that out of 26 cases, 13 were due to exogenous steroid treatment, three were due to significant trauma, and the rest due to other causes [8]. In our case report, the patient was healthy and had no co-morbidities or predisposing factors.

Patients usually present with sudden pain in the leg at the time of the injury; some report an audible snap and are unable to bear weight, which was also reported by our patient [9].

There are a number of diagnostic signs and tests for Achilles tendon rupture. Real-time high-resolution ultrasonography of the Achilles tendon is more sensitive than soft tissue radiography and gives dynamic images of the tendon [10]. The USG image shows heterogeneous hypoechoic areas with discontinuity and fluid-filled spaces, which is suggestive of a ruptured tendon.

Management options become complex with both non-operative and operative treatments being described in the literature for this unusual injury. Reconstruction options become a problem when there is a signiﬁcant delay and, more so, when the injury is bilateral.

Non-athletes may be treated non-operatively while operative treatment is the method of choice for athletes. We opted for operative intervention in the limb where the tendon was torn completely and gave conservative treatment for the limb where the tendon was partially torn.

Our patient achieved satisfactory healing as he was not on steroids, had no other risk factors, and had healthier tendons, which healed well.

Bilateral Achilles tendon rupture is associated with signiﬁcant morbidity because of rehabilitation problems after repair. Patients have difficulty with ambulation as plaster immobilization of both ankles is necessary to allow the injured tendon to heal. We allowed full weight-bearing for our patient after eight weeks of treatment after confirmation of complete healing of the tendons.

## Conclusions

Rupture of the Achilles tendon is relatively common in male athletes, although the bilateral rupture of the tendon is rare and poses a challenge for management. Hence, awareness about this entity is necessary for proper management of such cases.
